# Estrus induction and fertility response following different treatment protocols in Murrah buffaloes under field conditions

**DOI:** 10.14202/vetworld.2016.1466-1470

**Published:** 2016-12-22

**Authors:** L. Kumar, J. B. Phogat, A. K. Pandey, S. K. Phulia, S. Kumar, J. Dalal

**Affiliations:** 1 Department of Veterinary Gynaecology and Obstetrics, College of Veterinary Sciences, Lala Lajpat Rai University of Veterinary and Animal Sciences, Hisar - 125 004, Haryana, India; 2 Department of Veterinary Gynaecology and Obstetrics, Teaching Veterinary Clinical Complex, College of Veterinary Sciences, Lala Lajpat Rai University of Veterinary and Animal Sciences, Hisar - 125 004, Haryana, India; 3 Department of Animal Physiology and Reproduction, ICAR-Central Institute for Research on Buffalo, Hisar - 125 004, Haryana, India

**Keywords:** anestrus, Cosynch-plus, estrus induction, norgestomet

## Abstract

**Aim:**

The aim of this study was to evaluate the efficacy of three different treatment protocols for estrus induction and conception rate in postpartum anestrus buffaloes during breeding season under field conditions.

**Materials and Methods:**

The 47 postpartum anestrus buffaloes of the 2^nd^ to 6^th^ parity were divided into three groups. Group 1 (n=16): Buffaloes received cosynch treatment, that is, buserelin acetate 10 µg on day 0 and 9, cloprostenol 500 µg on day 7 followed by fixed-time artificial insemination (FTAI) at the time of second buserelin acetate and 24 h later. Group 2 (n=15): Buffaloes received norgestomet ear implant subcutaneously for 9 days, estradiol benzoate 2 mg on the day of implant insertion (day 0), pregnant mare serum gonadotropin (PMSG) 400 IU and cloprostenol 500 µg on day 9 followed by AI at 48 and 72 h after implant removal. Group 3 (Cosynch-plus, n=16): Buffaloes received Cosynch protocol as per Group 1 except an additional injection of PMSG 400 IU (i.m.) was given 3 days before the start of protocol and FTAI done at the same time of Group 1. Pregnancy diagnosis was performed after 45 days of AI.

**Results:**

The estrus induction response following the treatment was 81.3%, 100%, and 93.7% in Group 1, 2, and 3, respectively. The buffaloes of Group 1, 2, and 3 expressed intense (38.4%, 60% and 46.6%, respectively) and moderate estrus (46.1%, 26.6%, and 40%, respectively). The conception rates in Group 1, 2, and 3, at FTAI and overall including subsequent estrus were 37.5% and 62.5%, 53.3%, and 66.6%, 56.3%, and 75%, respectively.

**Conclusion:**

All the three treatment protocols can be effectively used for induction of estrus with acceptable conception rate in postpartum anestrus buffaloes during breeding season under field conditions. However, Cosynch-plus (similar to Cosynch protocol except addition of PMSG, 400 IU 3 days before the start of first buserelin acetate administration) protocol results comparatively better pregnancy rate.

## Introduction

The animal husbandry practices are vogue in most of the buffalo rearing countries including India are such that the single or few buffaloes are kept by individual farmers. Anestrus condition in buffaloes is considered the most frustrating and challenging reproductive problem responsible for economic losses to the farmer by decreasing milk production and net calf crop in a female’s lifetime. Estrus induction protocols using various hormones such as gonadotropin-releasing hormone (GnRH), progesterone (CIDR, Crestar), estradiol, pregnant mare serum gonadotropin (PMSG), and prostaglandins associated with fixed-time artificial insemination (FTAI) have been tried for the treatment of anestrus with variable success rates [1-5]. Progesterone in any form acts by maintaining a negative feedback over hypothalamus-pituitary axis and restricting the release of gonadotrophins, that is, follicle-stimulating hormone and luteinizing hormone, although the synthesis of gonadotropin still remains to continue. When the negative feedback is withdrawn, there is reflex release of these gonadotrophins resulting in induction of estrus within 2-5 days of progesterone removal [6,7]. Administration of PMSG and prostaglandin F2 alpha (PGF_2α_) at the time of progesterone withdrawal improves the conception rate in cows [2,8].

Further, the Ovsynch treatment – A sequence of GnRH - PGF_2α_ - GnRH injections became popular for estrus synchronization in cattle and buffalo over the last decade, resulting in fertility to timed AI that was similar to that of insemination after detection of estrus [9]. Cosynch is an alternative to Ovsynch protocol that eliminates one time animal handling by coinciding AI with second GnRH injection [10]. The diameter and stage of follicle development at first GnRH injection are important for efficiency of Cosynch protocol [11], particularly when follicle diameter is more than 9 mm size that results better conception rate [12]. It has been reported that follicle of diameter 10-12 mm was present at the time of first GnRH injection when PMSG was administered 3 days before Cosynch protocol [13].

Keeping in view the above information, we have chosen the three treatment protocols for treatment of anestrus condition in buffaloes. Moreover, so far most of the estrus induction trials in buffaloes with fixed time insemination have been conducted at organized farms. However, literature with the effectiveness of these protocols under field conditions is scanty where managemental conditions are entirely different and heat detection facilities are lacking. Thus, fixed-time insemination following estrus induction should overcome the problem of variable ovulation time and heat detection.

This study has been designed with the objective to investigate the efficacy of these three estrus induction protocols with respect to estrus induction and conception rate under field condition.

## Materials and Methods

### Ethical approval

The experiments on animals including all procedures of this study are carried out after approval by the Institutional Animal Ethics Committee.

### Location and selection of animals

The study was conducted on postpartum true anestrus Murrah buffaloes under field conditions in Hisar and Fatehabad districts of Haryana during the breeding season (September to February) when the environmental temperature ranges between 17 and 30°C. A total of 47 buffaloes (parity: 2^nd^ to 6^th^, body condition: 3 and above of 5 point scale) not detected in heat for 4 months after calving were included in the experiment. All the animals were kept chained to their place in villages, suckled and milked twice a day. The feeding practice followed was according to the availability of seasonal green fodder with required concentrates as per milk yield of the individual animal. The animals were having a history of normal parturition without any apparent sign of pathological condition of the reproductive tract. The anestrus condition was diagnosed by repeated per rectal examination of genitalia at an interval of 11-12 days; with smooth ovaries or without any palpable cyclic structure on either occasion. In addition, plasma progesterone concentration on the day of start of treatment (second occasion of rectal palpation) was measured using enzyme-linked immunosorbent assay kit which was <0.6 ng/ml in these animals confirmed the acyclicity.

### Experimental design

All the buffaloes synchronized for estrus between the month of September and February. The buffaloes were divided into three groups. Group 1 (n=16): Buffaloes received Cosynch treatment, that is, buserelin acetate 10 µg (i.m.) on day 0 and 9, cloprostenol 500 µg (i.m.) on day 7 followed by FTAI at the time of second buserelin acetate and 24 h later. Group 2 (n=15): Buffaloes received norgestomet ear implants subcutaneously for 9 days, estradiol benzoate 2 mg (i.m.) on the day of implant insertion (day 0), PMSG 400 IU and cloprostenol 500 µg (i.m.) on day 9 followed by AI at 48 and 72 h after implant removal. Group 3 (Cosynch-plus, n=16): Buffaloes received Cosynch protocol as per Group 1 except an additional injection of PMSG 400 IU (i.m.) was given three days before the start of protocol and FTAI done at the same time of Group 1. All the inseminations were performed by same person with frozen thawed semen of Murrah buffalo.

### Monitoring of animals for fertility response

The animal was observed daily for estrus induction up to 96 h from the day of cloprostenol administration. Estrus induction rate following different treatments was recorded by observing behavioral signs of estrus and rectal examination. The observers observed different behavioral estrus signs such as restlessness, bellowing, frequent micturition, tail raising, sniffing and/or licking of vulva, standing to be mounted by buffalo bull. Once any of these behavioral signs noticed then animal was checked for reddening of vulvar mucous membrane and further confirmed through rectal examination for uterine tonicity. Estrus intensity was recorded as intense, moderate, and weak as described by Layek *et al*. [14]. Pregnancy was confirmed per rectally after 45 days of AI. The fertility response of different treatment regimens were evaluated in relation to conception rate at FTAI and overall pregnancy rate including subsequent estrus.

### Statistical analysis

Chi-square test was used to compare the results of fertility response in different groups.

## Results and Discussion

The estrus induction response of Group 1, 2 and 3 buffaloes were 81.3%, 100%, and 93.7%, respectively (Table-1) and conception rates at FTAI were 37.5%, 53.3%, and 56.3% whereas overall pregnancy rate including subsequent estrus were 62.5%, 66.6%, and 75%, respectively (Table-1). The results were not statistically different significantly (p<0.05) among the groups. However, as per our expectations estrus induction rate was higher in Group-2 animals since the combined effect of progesterone priming of the brain for estradiol receptors and high endogenous estradiol with PMSG administration mainly responsible for behavioral signs [15]. Similarly, conception rate was better in Group 3 following Cosynch-plus treatment as PMSG injection 3 days before the first GnRH ensured sufficient follicle diameter [13] which essential for better conception rate [11,12].

**Table-1:** CR recorded at FTAI and subsequent AI in buffaloes treated with different protocols during breeding season.

Groups	Overall estrus induction rate (%)	CR after FTAI (%)	CR after subsequent AI (%)	Overall CR (%)
Group 1 (N=16)	13/16 (81.3)	6/16 (37.5)	4/10 (40.0)	62.5
Group 2 (N=15)	15/15 (100)	8/15 (53.3)	2/7 (28.5)	66.6
Group 3 (N=16)	15/16 (93.7)	9/16 (56.3)	3/7 (42.8)	75

CR=Conception rates, FTAI=Fixed time artificial insemination, AI=Artificial insemination

The results of Group 1 animals of present study are in agreement with the others following Ovsynch and Cosynch protocol who recorded estrus induction rate of 83-100% with 33.3-37.5% conception rate in cows [16] and anestrus buffaloes [17,18] and cyclic buffaloes [19,20] under farm conditions. However, slight lower estrus response (66.67%) with 33.33% conception rate following AI at induced estrus was observed by Tiwari *et al*. [21] in postpartum anestrus buffaloes during summer season. Seasonal variation may be responsible for poor results in later study.

As far as Group 2 is concerned, our findings are in agreement of other researchers who recorded a cent percent estrus induction response with similar conception rate (55-67%) following the fixed time AI in norgestomet plus PMSG treated anestrus buffaloes under farm conditions [22-24]. Similarly, under field conditions, observations of Naseer *et al*. [25] with 60% conception rate in CIDR+PMSG treated anestrus buffaloes during summer season again corroborate our findings. However, a slightly better conception rate (75%) with same protocol has been observed by Nayak *et al*. [26] in postpartum anestrus buffaloes under farm conditions during breeding season. The slightly better conception rate in later investigation compared to the present study might be attributed to different nutrition and management conditions at farm and field level. The better induction of estrus in norgestomet plus PMSG treated buffalo might be through the synergistic effect of endogenous and exogenous gonadotropin on folliculogenesis, oocyte maturation, and subsequent estradiol production [27].

Similar to Cosynch-plus protocol of current experiment in Group 3, a conception rate of 50-66% following Ovsynch-plus in buffalo heifers was also achieved by others [28,29]. However, Ovsynch-plus treatment resulted in estrus induction rate of 42-66% and pregnancy rate of 15-30% in buffalo heifers during summer season [30,13] which are much lower than our observations. The better estrus induction and conception rate in present investigation could be due to different season and parity between studies [4,11] apart from better care of individual animals for heat detection/management in field.

**Figure-1:**
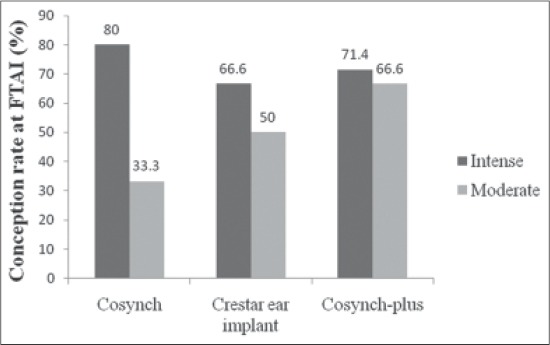
Comparative conception rate depending on intensity of estrus following different estrous synchronization protocols in buffalo.

When the intensity of estrus response was taken into an account following different treatment protocols, an intense estrus response of 38.4%, 60%, and 46.6%, and moderate estrus response of 46.1%, 26.6%, and 40%, was observed in Group 1, 2, and 3, respectively (Table-2). In category of intense and moderate estrus response a conception rates of 80% versus 33.3%, 66.6% versus 50%, 71.4% versus 66.6% was achieved in Group 1, 2, and 3, respectively (Figure-1). However, few animals (15.3%, 13.3%, and 13.3% in Group 1, 2, and 3, respectively) in each group exhibited weak signs of estrus and none of these buffaloes conceived following AI. The higher intense response in norgestomet plus PMSG treated animals in Group 2 compared to other groups might be due to the effect of exogenous progesterone, which sensitizes the hypothalamus receptors for the estrogen, and higher estradiol production by larger follicles in this group following PMSG injection mainly responsible for behavioral signs of estrus [23,26].

**Table-2:** Estrus response depending on intensity of heat symptoms following different estrus induction protocols in buffaloes.

Group	Estrus response			

	Overall estrus induction rate (%)	Intense estrus (%)	Moderate estrus (%)	Weak estrus (%)
Group 1	13/16 (81.3)	5/13 (38.4)	6/13 (46.1)	2/13 (15.3)
Group 2	15/15 (100)	9/15 (60)	4/15 (26.6)	2/15 (13.3)
Group 3	15/16 (93.7)	7/15 (46.6)	6/15 (40)	2/15 (13.3)

As far as the conception rate based on the intensity of estrus response is concerned, the results of this study revealed that pregnancy rate was higher in animals showing intense than moderate heat signs in all the three groups. The higher pregnancy rates in animal showing intense response might be due to the appropriate size achieved by follicles and their ovulation within the time window of FTAI compared to moderately responding animals. However, the results did not vary significantly (p<0.05) between the groups perhaps due to the less number of animals in intense and moderate category in each group. The findings of the current study with conception rate of 67-80% are in agreement with that of earlier observations by Nayak *et al*. [26] who achieved a conception rate of 75-100% in animals showing intense heat response.

## Conclusions

All the three treatment protocols can be effectively used for induction of estrus with acceptable conception rate in postpartum anestrus buffaloes during breeding season under field conditions. However, Cosynch-plus (similar to Cosynch protocol except addition of PMSG, 400 IU 3 days before the start of first buserelin acetate administration) protocol results comparatively better pregnancy rate.

## Authors’ Contributions

This experiment was conducted by LK under guidance of JBP during his master degree. AKP and SKP were involved in data analysis and manuscript drafting. SK and JD were indulged during conduct of experiment and hormonal analysis.
